# Analysis of the application of policy instruments for close-knit county medical communities based on Hood’s classification

**DOI:** 10.3389/fpubh.2025.1552590

**Published:** 2025-03-06

**Authors:** Huang Xianlu, Feng Lei

**Affiliations:** ^1^College of Public Health, Chongqing Medical University, Chongqing, China; ^2^College of Marxism, Chongqing Medical University, Chongqing, China

**Keywords:** close-knit county medical communities, policy instruments, content analysis, Hood’s classification, Chongqing municipality

## Abstract

**Objective:**

By analyzing the selection and application of policy instruments in the construction of close-knit county medical communities in Dazu District, Chongqing, this study aimed to propose countermeasures to optimize these policy instruments, offering valuable lessons for the construction of close-knit county medical communities.

**Methods:**

Based on Hood’s classification, an analytical framework was constructed that incorporated the selection of basic policy instruments (X dimension) and the different phases in the construction process (Y dimension). Nvivo was used to analyze policy documents (2015–2023) related to the construction of close-knit county medical communities in Dazu District.

**Results:**

In the X dimension, the nodality-based instruments accounted for 14.3%, the authority-based instruments accounted for 34.2%, the treasure-based instruments accounted for 16.4%, and the organization-based instruments accounted for 35.2%. In the Y dimension, the total number of policy instruments used in Dazu District across the following three phases—policy initiation, piloting, and promotion—showed an increasing trend, aligning with the number of policies. These statistics indicated several issues such as limited use of nodality-based and treasure-based instruments, an incomplete policy assessment indicator system, insufficient policy synergy and incentives, and a lack of operationalization of strategic measures.

**Conclusion:**

The government should increase the use of nodality-based and treasure-based instruments, improve the assessment indicator system, strengthen policy synergy mechanisms and incentives, and optimize the structure of talent resources to improve the operability of the policies.

## 1 Introduction

County health plays a crucial role in China’s medical and healthcare development, acting as a bridge. Promoting the hierarchical medical system within counties has become a top priority. The close-knit county medical communities are one of the main organizational models for exploring the hierarchical medical system. These communities, consisting of a rural medical consortium led by county-level hospitals, include township and village medical institutions, as well as other healthcare organizations. The goal is to achieve commonality in services, interests, responsibilities, and development across all levels of medical institutions. This structure is designed to promote cooperation among healthcare providers, enhance the in-county consultation rate and patient satisfaction, and ensure control over medical costs and improvement in the quality of care.

In September 2018, Chongqing launched the pilot construction of medical communities, with Dazu District being one of the selected areas. Starting in January 2019, two hospital groups were formed, led by Dazu District People’s Hospital and Dazu District Hospital of Traditional Chinese Medicine, with 11 township health centers as members. By April 2020, three close-knit medical communities had already been established in the region. Since the pilot began, the results of the hierarchical medical system have become evident. The consultation rate in Dazu District has surpassed 90%, and the proportion of visits to primary-level medical institutions has reached 67.7%, marking an increase of 16.9 percentage points compared to the period before the pilot program. The awareness rate of basic public health services has also improved, rising from below 60% before the reform to 76.17% (1). Various reform measures, including the mutual recognition of medical tests, upgrades to medical institutions’ information systems, and optimization of performance distribution systems, have achieved positive results (2). By December 2023, the construction of close-knit county medical communities in China had entered a stage of comprehensive promotion.

Given the relatively short existence of close-knit county medical communities, scholars have predominantly focused on experiential studies related to their construction progress (3), operational status (4), and outcome evaluation (5). There is a lack of research on policy texts and their effects on close-knit county medical community construction, which involves communication and collaboration across different levels of medical institutions and requires policy support and intervention. Therefore, this study analyzes the policy texts and employs Hood’s classification to conduct a policy science assessment from the perspective of policy instruments. It proposes targeted recommendations for optimizing the selection of policy instruments to support the construction of close-knit county medical communities.

## 2 Materials and methods

### 2.1 Sources of materials

Using the keywords “the hierarchical medical system,” “integrated healthcare,” “primary healthcare system,” and “close-knit county medical communities,” official policy documents were searched on the government websites of China (Chongqing /Dazu District) and the National Health Commission, as well as in the databases of Peking University Law Info (Pkulaw) and CNKI. A total of 126 policy documents were obtained through full-text searches using the above keywords. To ensure the representativeness of the research subjects and the accuracy of the research results, the following criteria for policy inclusion were established: (1) The policy type must include laws, regulations, work plans, notices, and reply letters in the form of textual documents. (2) The policy text must directly mention close-knit county medical communities or reflect the corresponding policy objectives of the government. (3) The policy must be issued by an authoritative official institution. Based on these criteria, 78 policy documents were selected from 2015 to 2023, including 25 national-level policy documents, 23 Chongqing municipal-level policy documents, and 30 Dazu district-level policy documents (Table 1).

**Table 1:** Policy documents related to the construction of close-knit county medical communities.

Form	Release time	Name of the policy
China	2019.5.15	Notification on promoting the construction of close-knit county medical and healthcare communities
	2020.8.31	Notification on the issuance of assessment criteria and monitoring indicator system for the construction of close-knit county communities (for trial implementation)
	2023.12.30	Guiding opinions on comprehensively promoting the construction of close-knit county medical communities
	……	
Chongqing	2017.7.21	Implementation opinions on promoting the construction and development of medical consortia
	2020.1.19	Specific work program on the county medical community construction
	2023.10.18	Notification on the issuance of the implementation plan for further improving the medical and healthcare service system in chongqing
	……	
Dazu District	2017.7.13	Notification on issuing the key tasks of chongqing dazu District in deepening the reform of the medical and health system in 2017
	2018.12.29	Notification on the issuance of the implementation plan for the improvement of modern hospital management system in Dazu District, Chongqing
	2020.5.7	The issuance of the “Work plan for the medical construction of connectivity in medical community” “Work plan for the construction of personnel connectivity in medical community” “Work plan for financial support and fiscal connectivity in medical community” “Reform plan for medical insurance fund payment methods in medical community” “Implementation plan for performance evaluation of management in medical community”
	2022.2.14	Notification on the issuance of performance assessment and evaluation measures for member units and principal officers of the medical community
	2023.9.11	Notification on the issuance of the implementation plan for the improvement of the medical insurance and relief system for serious and major diseases
	……	

### 2.2 Research methods

Content analysis is a research method that systematically examines and interprets textual content to identify patterns, themes, and meanings. In this study, we primarily employed the Nvivo software to conduct a quantitative analysis of the selected policy texts (6). Nvivo is qualitative data analysis software commonly used for processing textual data. Given the large volume and complex structure of policy texts, Nvivo offers flexible coding methods to quantify specific policy items and identify trends in policy changes or shifts in policy priorities through its visualization tools. In this study, we collected policy texts closely related to the construction of close-knit county medical communities and used word frequency statistics and coding functions to conduct comprehensive queries, node-by-node coding, statistical analysis, and graphical presentations of all texts.

First, theme terms closely related to close-knit county medical communities were extracted from specific policy articles, and the policy instruments applied within them were integrated and summarized into corresponding sub-nodes. Then, the classification of the policy instruments was set as the first-level dimension (tree nodes), while the policy instruments summarized under each theme were set as the second-level dimension (sub-nodes). The coded content was then categorized under the corresponding sub-nodes, with each policy content treated as a unit. Finally, the text content within each node was adjusted and improved, and the coding results were organized and statistically analyzed.

## 3 Analytical framework construction

This study constructed a textual analysis framework for the policy of close-knit county medical communities in Dazu District, examining it from two dimensions: policy instruments (X dimension) and construction process (Y dimension).

### 3.1 Dimension X: selection of basic policy instruments

Driven by the dual forces of economic globalization and the information technology revolution, the study of tools for improving performance and transforming government governance has emerged. The study of policy instruments became a key focus in public policy research in the West after the 1980s. As a means for governments to achieve their objectives, policy instruments serve as the linkage mechanism between policy goals and actions. They are also the approaches adopted by governments to meet public needs (7).

This study used Hood’s classification to organize policy instruments. Christopher C. Hood, a famous British scholar, argued that governments deal with public problems by using four broad “governmental resources,” namely nodality, authority, treasure, and organization. This leads to a systematic classification framework that classifies all policy instruments into four corresponding types: “nodality-based instruments,” “authority-based instruments,” “treasure-based instruments,” and “organization-based instruments.” (8) This classification breaks away from the traditional limitations of “degree of coercion” or “form of intervention” as standards, focuses on the essential attributes of government governance resources, and emphasizes the selection of policy instruments that align with the government’s resource endowment and the policy environment, providing a good foundation for analyzing the motivation behind policy instrument selection in the construction of close-knit county medical communities. After using Hood’s classification to roughly sort the policy texts, the theme words were set as sub-nodes, named after specific policy instruments. These were then summarized according to the definition of each category (Table 2).

**Table 2:** Definition of each classification and corresponding specific policy instruments (sub-nodes).

Classification	Definition	Sub-nodes
Nodality-based instruments	Promoting the integration and standardization of management through information sharing.	Mutual exchange standardization publicity and promotion cultural construction
Authority-based instruments	Employing mandatory directives for coordinated planning, conducting assessments, and ensuring the effectiveness of the construction outcomes.	Target planning personnel management operating mechanism performance appraisal supervision and management policy support resource allocation
Treasure-based instruments	Promoting the flow of resources and integration of services through economic means and other interest-related approaches.	Service prices remuneration system medical insurance payment government investment financial management industry linkage commercial insurance
Organization-based instruments	Organizations strengthen their construction to facilitate the implementation of policies. On the other hand, fully utilizing social forces, resources, and technologies can achieve policy objectives.	Functional positioning service expansion digital platform talent cultivation drugs and consumables technical expertise organizational safeguards non-governmental medical services

### 3.2 Dimension Y: different phases in the progress of the construction

After sorting out the overall process of the construction of close-knit county medical communities in Dazu District, this study divided the collection of policy documents from 2015 to 2023 into three different phases and explored the characteristics of the policy instruments in each of these three phases. The first stage was policy initiation (2015–2017), which put forward the goal of promoting the construction of healthcare consortia as part of the general direction of healthcare system reform. However, there were no other detailed implementation plans and supporting policies. The second stage was the policy pilot phase (2018–2020), which put forward the implementation of the “two-step” policy in June 2018. Dazu District was identified as a national pilot district in August 2019, during which a series of supporting documents were issued. The third stage was policy promotion (2021–2023). In February 2021, specific policy guidance was launched by the Chongqing municipal government, and Dazu District issued several policy documents to comprehensively promote the construction of close-knit county medical communities.

### 3.3 Reliability check

To ensure the accuracy and credibility of the coding, this study was independently completed by two coders who coded the policy text. Any discrepancies in the coding results were discussed and resolved. The Kappa consistency coefficient was used to test the consistency of the coding, and finally, the Kappa coefficient value was greater than 0.810. The coding in this study showed a certain degree of stability and reliability.

## 4 Results and discussion

### 4.1 Basic selection and application of the policy instruments

The statistical results after the coding (Table 3), according to Hood’s classification, showed that 14.3% of the policy instruments were nodality-based, 34.2% were authority-based, 16.4% were treasure-based, and 35.2% were organization-based. This shows that the construction of close-knit county medical communities in Dazu District primarily relies on the use of authority-based and organization-based instruments and that the use of nodality-based and treasure-based instruments is relatively limited.

**Table 3:** Coding and percentage of the policy instruments based on Hood’s classification.

Classification	Policy instruments(sub-nodes)	Number of source documents	Percentage (%)	Coded reference points	Percentage (%)
Nodality-based instruments	Mutual exchange Standardization Publicity and promotion Cultural construction	42 53 51 27	54 68 65 35	66 106 102 36	3.0 4.9 4.7 1.7
	Subtotal	72	92	310	14.3
Authority-based instruments	Target planning Personnel management Operating mechanism Performance appraisal Supervision and management Policy support Resource allocation	56 42 56 37 62 35 29	72 54 72 47 79 45 37	110 85 206 67 150 63 62	5.1 3.9 9.5 3.1 6.9 2.9 2.9
	Subtotal	75	96	743	34.2
Treasure-based instruments	Service prices Remuneration system Medical insurance payment Government investment Financial management Industry linkage Commercial insurance	31 34 43 51 31 6 12	40 44 55 65 40 8 15	54 47 76 80 59 27 13	2.5 2.2 3.5 3.7 2.7 1.2 0.6
	Subtotal	65	83	356	16.4
Organization-based instruments	Functional positioning Service expansion Digital platform Talent cultivation Drugs and consumables Technical expertise Organizational safeguards Non-governmental medical services	29 51 55 50 44 36 62 35	37 65 70 64 56 46 79 45	68 143 101 91 101 68 121 73	3.1 6.6 4.6 4.2 4.6 3.1 5.6 3.4
	Subtotal	77	99	766	35.2

The nodality-based instruments were the least used. The limited use of informational policy instruments may cause problems such as delayed information deployment, inappropriate coordination guidelines, and a confusing policy environment. These challenges, in turn, adversely affect the policy framework of close-knit county medical communities. The highest proportion of emphasis on “Standardization” reflects the government’s priorities, with as many as 106 coded reference points covering five key aspects—"administration, personnel, finance, medical care, and public health”—which specify measures for unified management (1). In addition, the government has frequently used “publicity and promotion” tools to consolidate the results of the construction of close-knit medical communities, accounting for 4.7% of the total.

The authority-based instruments accounted for the second-largest proportion. The main body of the policy primarily focuses on ensuring the efficiency of its implementation through direct intervention, such as “supervision and management” and other tools based on relevant rules and regulations, which accounted for approximately 6% of the secondary classification. The number of coded reference points for “operating mechanism” was as high as 206, accounting for 9.5%, indicating that improving the planning and implementation mechanism of the healthcare service system is the main way to build a close-knit county medical community in Dazu District. Although authoritative policy instruments can improve implementation efficiency among the members of the medical community and accelerate the operation and coverage of measures, over-reliance on rigid tools may also result in weak motivation among the members of the medical community, low public acceptance, and other undesirable consequences.

The use of the treasure-based instruments was relatively low, and the secondary categories were more evenly distributed. Among them, the proportion of “government investment” was the highest, reaching 3.7%. “Health insurance payment” was slightly less than the former, accounting for 3.5%. Specific measures, such as the implementation of the DRG payment method, the implementation of the total budget, and total liquidation, were often mentioned in the policy documents. The use of incentive-type instruments in the secondary classification was relatively low, and the treasure-based instruments related to the “remuneration system” accounted for only approximately 3% of the total, which will inevitably lead to a lack of strength due to insufficient resource support during the process of policy implementation.

The organization-based instruments were used the most, slightly more than the authority-based instruments. The concept behind the construction of close-knit county medical communities is to first strengthen the primary healthcare system and then drive the development of medical health in the county (9). Organization-based instruments help strengthen the functionality of medical institutions within the county medical community. The government often uses sub-node policy instruments, such as “cultivation of talent,” to improve the level of primary healthcare technology. In addition, medical technicians regularly visit member units for consultations, room inspections, and surgical guidance (10). It is also worth mentioning that the “digital platform” contained 101 coded reference points, accounting for 4.6% of the total, demonstrating the deepening development of “Internet + healthcare.” By 2023, Dazu District had established an information platform integrating electronic medical records from medical institutions, electronic health records (EHRs), and population and family databases (11). However, the excessive use of organization-based instruments may cause medical institutions to focus solely on their development, without exploring the collaborative mechanism. This could lead to a fragmented situation, known as the “Silo Effect,” which contradicts the original purpose of the construction of close-knit medical communities.

### 4.2 Application of the policy instruments in progress

In terms of the number of policy releases, among the 78 policy documents included in the study, 19 were released in 2015–2017, 25 in 2018–2020, and 34 in 2021–2023, showing a significant increase in the number of county healthcare policies introduced in Dazu District. To further clarify the use and characteristics of the policy instruments at each stage, the 78 policy documents were matrix coded using Nvivo, with “rows” representing Hood’s classification and “columns” representing the three phases of the process. This method was used to obtain the results of the advancement phases and the nodes of the policy instruments (Table 4).

**Table 4:** Number of coded reference points for the policy instruments at each phase.

	Initiation: 2015–2017	Pilot: 2018–2020	Promotion: 2021–2023
Treasure-based	126/22.0%	85/15.3%	92/15.7%
Organization-based	179/32.4%	200/34.1%	262/39.6%
Authority-based	190/33.9%	205/37.1%	264/34.6%
Modality-based	64/11.6%	95/13.5%	127/10.1%
total	559	585	745

As shown in the table, the total number of policy instruments used in Dazu District during the three phases—policy initiation, pilot, and promotion—showed an increasing trend, consistent with the increase in the number of policies. This indicates that as the direction of the reform becomes gradually clearer and the policy objectives more defined, the policy measures become more refined and intensive.

During the policy initiation period, the use of the treasure-based instruments was more significant than in the other two phases, with “health insurance payment” occupying an irreplaceable position in the early reform of the healthcare system. In 2017, a policy released by the municipal government stated: “Select 3–5 medical communities to explore the implementation of various payment methods, such as total health insurance payment and other models of division of labor collaboration.” The guiding role of the medical insurance payment method in influencing the supply and demand of healthcare services has been repeatedly mentioned.

During the pilot phase, the gap between the treasure-based instruments and nodality-based instruments narrowed, while the dominance of the authoritative policy instruments was fully demonstrated. In January 2020, the Chongqing municipal government issued a work program for the construction of a county medical community, and Dazu District released five supporting policies in May. These policies outlined a clear “target planning” and “operating mechanism,” providing detailed regulations and adjustments. Specific indicators were also established through measures such as “performance evaluation” and “supervision and management” to ensure the implementation of the policies, significantly increasing the proportion of authority-based instruments.

In the policy promotion stage, the organization-based instruments accounted for a larger proportion of the policy content, following the principles of improving the medical capacity of primary-level hospitals before enhancing the overall level of health services in the county. In August 2022, the government issued an implementation plan to promote the high-quality development of public hospitals, which led to the overall enhancement of county-level medical and health services. It can be predicted that organization-based instruments aimed at improving service capacity will continue to play a role for a long time in the future.

## 5 Problems and suggestions

Through the compilation and analysis of the current situation of close-knit county medical community construction in Dazu District, we can conclude that, compared to other countries, the advantage of China’s county medical community construction lies in its government-led integration ability, which can quickly mobilize resources through the support of a strong administrative system. In addition, China has a large population base and a high population density within counties, which make service coverage broader and more efficient after resource integration. Furthermore, China’s development of information technology is at the forefront globally. For example, telemedicine and electronic health records are increasingly used in county medical communities, providing a platform for information sharing and collaborative services. However, with the gradual progress of the policy, some issues are gradually revealed.

### 5.1 Problems

#### 5.1.1 Less use of nodality-based and treasure-based instruments

The results of the policy analysis showed that the proportion of authority-based and organization-based instruments is relatively high, indicating that the government’s approach to policy formulation may be influenced by rigid thinking and path dependence. The lack of nodality-based and treasure-based instruments makes it difficult to achieve policy coordination and ensure seamless integration across different policy cycles, which is bound to affect the operation of the entire medical community. Currently, China relies on government investment for the use of treasure-based instruments, while some developed countries, such as Germany, have a more complete financial support system for healthcare, with larger and more stable investment scales. For example, Germany has set up an annual total of 300 million euros in the Innovation Funds for the integration of healthcare delivery systems (12). In terms of using nodality-based instruments, Singapore has achieved broader clinical integration collaboration and information sharing through the medical group model, while maintaining the mechanism of healthy competition (13). In contrast, China’s county medical communities still suffer from insufficient information sharing and system integration, and medical information platforms in some of the regional medical communities have not yet been fully covered, resulting in the inability to share medical resources in real time.

#### 5.1.2 Incompleteness of the policy assessment indicator system

The results of the policy analysis showed that the distribution of sub-nodes policy instruments, which often correspond to assessment indicators, is uneven. Although terms such as “assessment” and “standards” from the content appear more frequently in various policy documents, their system is relatively incomplete. Moreover, the assessment indicators of China’s county medical communities are more focused on the downscaling of medical resources, improving service capacity, and the effectiveness of medical insurance funds. In contrast, developed countries such as the United Kingdom (UK) are more focused on managing the entire process of medical services and patient experience, including quality control across all stages—from disease prevention and diagnosis to treatment and rehabilitation (14).

#### 5.1.3 Policy synergy mechanisms and incentives need to be strengthened

Close-knit county medical communities involve medical organizations at all levels. However, the proportion of “non-governmental medical services” in the sub-nodes policy instruments was found to be only 3.4%, indicating that social medical organizations are not fully involved. On the contrary, Germany promotes the construction of medical associations through socialization and introduces an internal market to optimize the allocation of resources (15). Meanwhile, the policy system in China lacks positive incentives for medical personnel within the medical community, and the distribution mechanism is rigid. It is reported that the average annual income of rural doctors (about 60,000 yuan) is only 1/3 of that of doctors in county hospitals, leading to difficulties in talent retention. In contrast, in the UK, the remuneration of GPs can be up to 85% of that of specialists. As a result, these disincentives hinder the establishment of a sense of participation, mission, and motivation among the members (16).

#### 5.1.4 Lack of operationalization of strategic measures in some policies

Strategic measures refer to the use of purposeful and targeted policy instruments to refine policy objectives and implement them. They aim to break through bottlenecks during the policy platform period and solve unexpected situations flexibly. Their smooth implementation is closely related to talent resources. Currently, there is a structural shortage of primary healthcare talent in China. The number of practicing physicians per 1,000 people in counties (2.2) is still lower than that in cities (3.8), and the proportion of senior titles is less than 15%, which is significantly lower than the proportion of specialists among community doctors in Germany (more than 60%) (17). Facing the bottleneck of talent resources, the sub-nodes policy instruments of “talent cultivation” rarely mention targeted supply, and the proportion of “professional technical” is relatively low, which will inevitably lead to a lack of operationalization within the strategic policy system.

### 5.2 Suggestions

#### 5.2.1 Increasing focus on rationalizing the structure of policy instruments when designing policy frameworks

The government needs to strengthen the use of nodality-based and treasure-based instruments in the subsequent process. First, nodality-based policy instruments can enhance the understanding and recognition of the policy among the target group, thus effectively improving the policy environment (4). The GP system in the UK relies heavily on informational management, using electronic health records (EHRs) and electronic medical record systems (EMRSs) to achieve comprehensive management and sharing of patient information (18). As for EHRs, the Chinese government should improve the “digital platform” for primary healthcare, enable the digital storage and transmission of examination data, and gather such data in the cloud to lay the foundation for the widespread application and mutual recognition of medical data (19). Second, the government can draw on the Sanming municipality (20), a pilot city that the government has vigorously promoted, to optimize treasure-based instruments such as joint price-limit purchasing, monitoring of fee increases, and adjustment of service prices. Overall, the special fund model of Germany’s Innovation Funds can be borrowed to centralize resources for medical communities, avoiding the fragmented use of financial resources and improving efficiency (12).

#### 5.2.2 Improving policy assessment indicators and adhering to the orientation of health needs

The policy objective of close-knit county medical communities is to adhere to a people-centered health approach and, through the rational allocation of medical resources, to make sure that patients and residents within the county receive equitable health services. For example, the NHS Outcomes Framework in the UK covers a wide range of health-related evaluation indicators, including public health, social support, and determinants of health (21). This demonstrates a health outcome orientation characterized by multidimensionality, short-term safety, and long-term functionality, which enables public health, rehabilitation, and nursing organizations to better perform their professional roles. The Chinese government can strengthen the integration of services between medical communities, nursing institutions, and care institutions by following the example of Germany’s Regional Medical Alliance, which has passed legislation requiring hospitals and community clinics to divide and coordinate their work (22). It is helpful for strengthening the binding force of the system and for promoting close-knit county medical communities in the areas of “service expansion” and “industry linkage.”

#### 5.2.3 Strengthening the synergistic mechanism of policies and utilizing the incentives of policy instruments

The UK ensures an orderly flow of patients between general practitioners and specialists through a strict “gatekeeper system” (23). China can learn from this model to further improve the tiered diagnosis and treatment system, clarify the functional positioning of medical institutions at all levels, strengthen the role of general practitioners as “gatekeepers,” optimize the two-way referral process, guide the reasonable triage of patients, and promote the rational use of medical resources. At the same time, the government could learn from the performance incentive mechanism of Accountable Care Organizations(ACOs) in the United States (US) to optimize the internal distribution mechanism of county medical communities, thereby stimulating the enthusiasm of medical personnel (24). Moreover, it could improve the primary-level talent mobility system by providing effective incentives and clear promotion channels. In addition, the remuneration system needs to be reformed, with a focus on increasing the proportion of labor value and rationally arranging it to stimulate the enthusiasm of medical workers.

#### 5.2.4 Optimizing the structure of primary-level talent and improving the operability of policies

Japan’s community-based integrated care system has been implemented more successfully at the primary level of healthcare, improving the efficiency of primary health services by training specialized community nursing personnel (25). It is enlightening for the Chinese government to strengthen the training of talents in the county, improve the nursing service capacity of primary care organizations, and promote the diversified development of services. Cultivating talent to achieve the goal of downscaling medical resources not only enhances the service level of primary healthcare institutions but also helps address the patient trust crisis. China’s current referral rate of tertiary hospitals within the county is only 35%, which is significantly lower than the 80% local first-visit rate under Japan’s “regional healthcare linkage” system (26). Cultivating primary-level talent is more conducive to enhancing the public’s trust in primary care, thus improving the operability of the policy.

## Data Availability

The original contributions presented in the study are included in the article/supplementary material, further inquiries can be directed to the corresponding author.
